# Functional assessment of bioprosthetic mitral valves by cardiovascular magnetic resonance: *An* in vitro *validation and comparison to Doppler echocardiography*

**DOI:** 10.1186/s12968-020-00635-x

**Published:** 2020-07-30

**Authors:** Dimitrios Maragiannis, Matthew S. Jackson, Kyle Autry, Jose H. Flores Arredondo, Constantina Aggeli, Dimitrios Tousoulis, William A. Zoghbi, Dipan J. Shah, Stephen H. Little

**Affiliations:** 1 Department of Cardiology, 401 General Military Hospital of Athens, Leoforos Mesogion 138, 11525 Athens, Greece; 2 Department of Cardiology, Houston Methodist Hospital, Houston, TX USA; 3 1st Department of Cardiology, National and Kapodistrian University of Athens, Hippokration General Hospital, Athens, Greece

**Keywords:** Cardiovascular magnetic resonance; bioprosthetic valve, Mitral valve, Doppler echocardiography, Prosthetic valve stenosis

## Abstract

**Background:**

A comprehensive non-invasive evaluation of bioprosthetic mitral valve (BMV) function can be challenging. We describe a novel method to assess BMV effective orifice area (EOA) based on phase contrast (PC) cardiovascular magnetic resonance (CMR) data. We compare the performance of this new method to Doppler and in vitro reference standards.

**Methods:**

Four sizes of normal BMVs (27, 29, 31, 33 mm) and 4 stenotic BMVs (27 mm and 29 mm, with mild or severe leaflet obstruction) were evaluated using a CMR- compatible flow loop. BMVs were evaluated with PC-CMR and Doppler methods under flow conditions of; 70 mL, 90 mL and 110 mL/beat (*n* = 24). PC-EOA was calculated as PC-CMR flow volume divided by the PC- time velocity integral (TVI).

**Results:**

PC-CMR measurements of the diastolic peak velocity and TVI correlated strongly with Doppler values (*r* = 0.99, *P* < 0.001 and r = 0.99, *P* < 0.001, respectively). Across all conditions tested, the Doppler and PC-CMR measurement of EOA (1.4 ± 0.5 vs 1.5 ± 0.7 cm^2^, respectively) correlated highly (*r* = 0.99, *P* < 0.001), with a minimum bias of 0.13 cm^2^, and narrow limits of agreement (− 0.2 to 0.5 cm^2^).

**Conclusion:**

We describe a novel method to assess BMV function based on PC measures of transvalvular flow volume and velocity integration. PC-CMR methods can be used to accurately measure EOA for both normal and stenotic BMV’s and may provide an important new parameter of BMV function when Doppler methods are unobtainable or unreliable.

## Background

The functional evaluation of a bioprosthetic mitral valve (BMV) can at times be very challenging. Clinical guidelines recommend Doppler echocardiography as the initial method to assess BMV function [1, 2]. However, for some patients, the Doppler assessment of BMV function can be technically limited or unreliable. The use of cardiovascular magnetic resonance (CMR) to measure flow volume is well established, however a method to integrate flow velocities across a prosthetic valve and to derive an effective orifice area (EOA) has not been previously reported. The aim of this study was to evaluate the feasibility and accuracy of a novel phase contrast (PC)-CMR method to derive EOA for a range of normal and stenotic BMVs and to compare those values against in vitro and Doppler echocardiography standards.

## Methods

### Experimental model

**Fig. 1:**
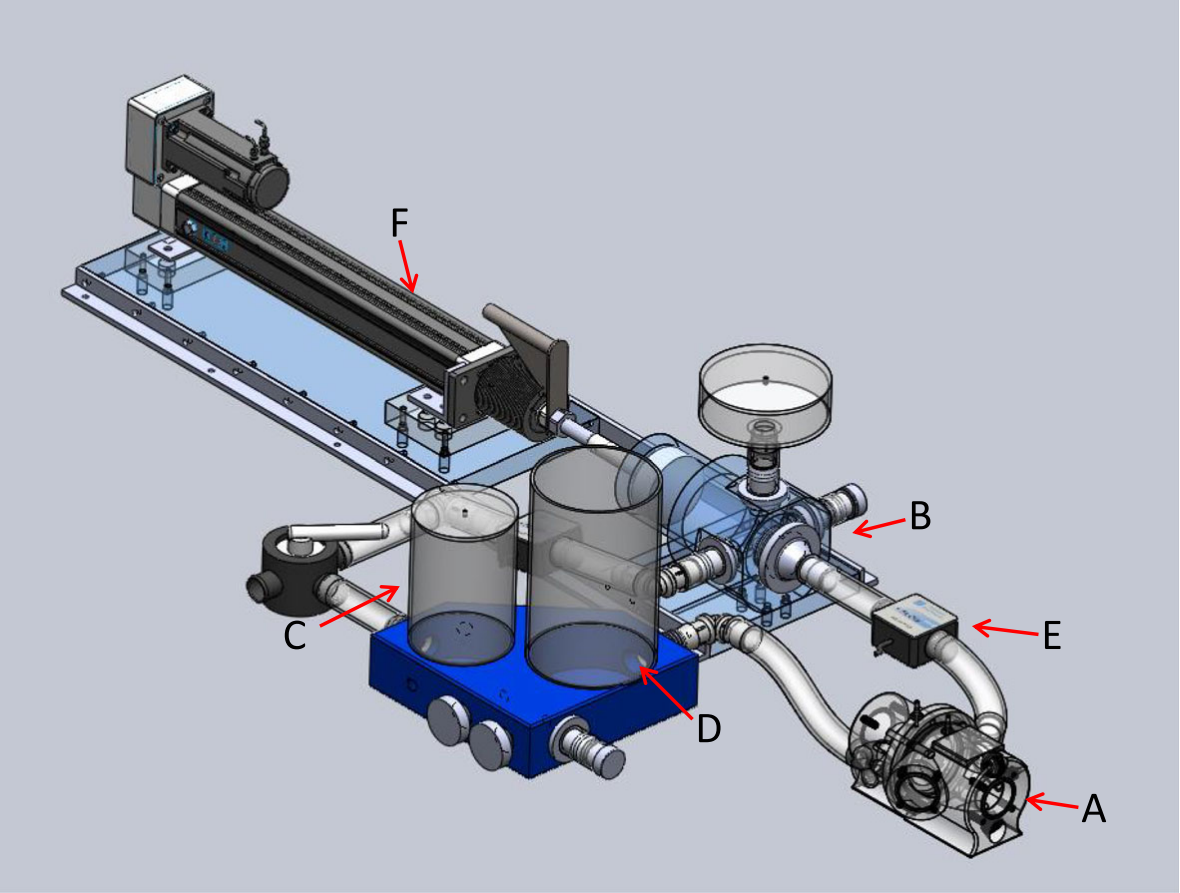
The circulatory loop. A mock ventricle and heart valve imaging chamber **a**, pulsatile pump **b**, arterial compliance/resistance elements **c**, fill reservoir **d**, high-fidelity flow transducers **e** and the linear actuator **f**

**Fig. 2:**
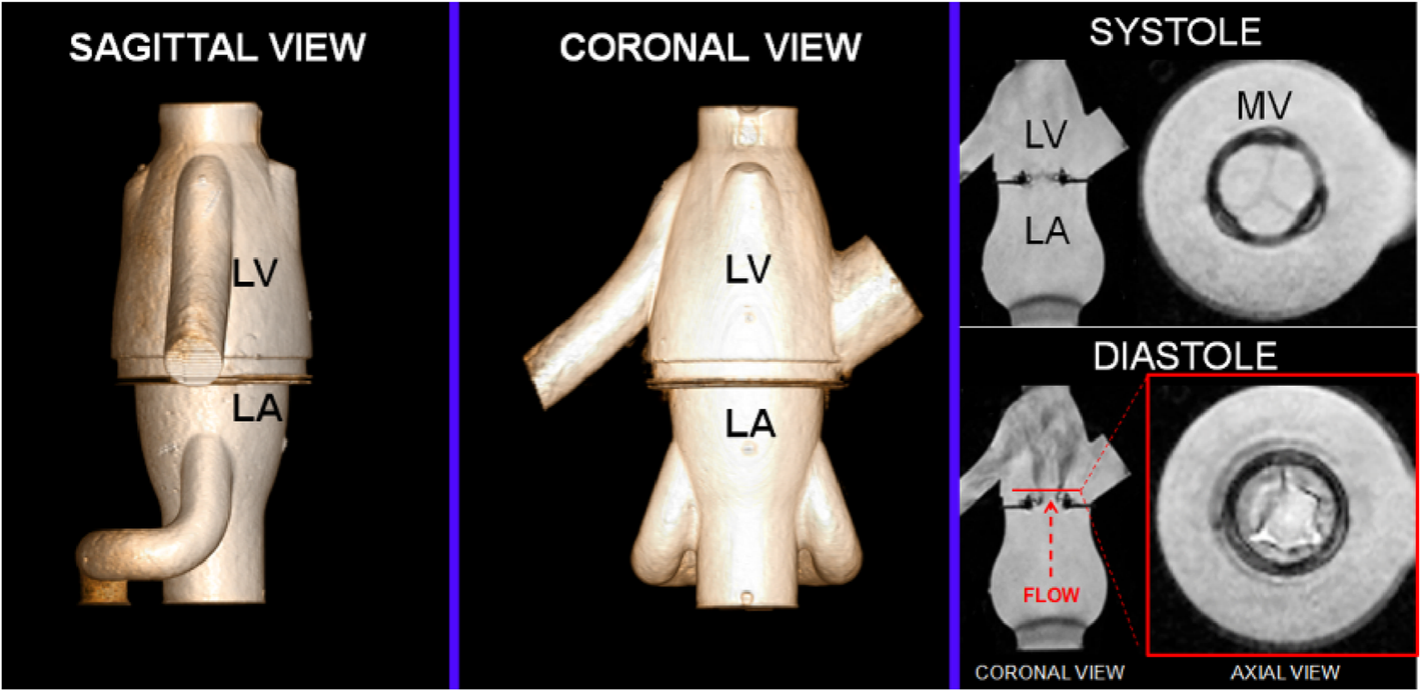
Cardiovascular magnetic resonance (CMR) images of the mock ventricle. Cine CMR images (*right*) depict systolic and diastolic function of a 27 mm bioprosthetic mitral valve (BMV)

**Fig. 3:**
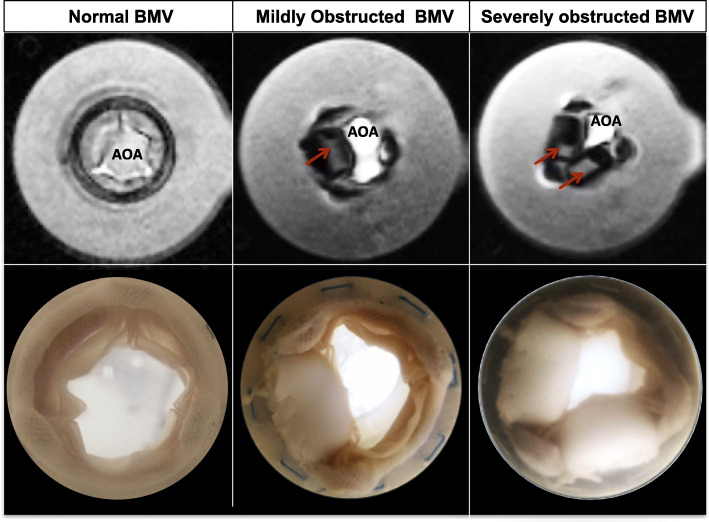
Normal and obstructed bioprosthetic mitral valves. Axial cine CMR images (*above*) and photographs (*below*) of normal 29 mm BMV, a valve with single leaflet obstruction, and a valve with double leaflet obstruction

Our CMR-compatible in vitro flow loop has been previously described [3]. In brief, the circulatory loop includes a pulsatile pump, an imaging chamber consisting of a mock ventricle and atrium separated by a bioprosthetic valve, and ultrasound imaging windows simulating clinical Doppler echocardiography windows (Fig. 1). The modular imaging chamber (atrium and ventricle) permits functional imaging of any prosthetic mitral valve under tailored hemodynamic flow conditions. The entire flow loop was constructed from non-ferromagnetic materials (Fig. 2). An inline flow transducer (Transonic Systems Inc., Ithaca, New York, USA) was used to measure transvalvular flow. Pressure transducers (Millar Instruments, Houston, Texas, USA) placed in the atrium and ventricle measured trans-mitral diastolic pressure. Compliance and resistance elements were used to further refine hemodynamic parameters. The system was filled with 4.5 L of a blood analog of 30% glycerin, 70% water, and 0.01% cornstarch [4]. To mimic BMV obstruction due to pannus ingrowth or thrombus, we modeled mild BMV stenosis by suturing a single silicone rubber occluder to the ventricular aspect of the BMV sewing ring between two of the BMV struts. In this way a single occluder limited the diastolic motion of a single leaflet. Severe obstruction was modeled using a second occluder affixed in a similar fashion to obstruct diastolic motion of a second leaflet (Fig. 3).

### Cardiovascular magnetic resonance

Four different sizes BMVs (Mosaic 27, 29, 31 and 33 mm; Medtronic, Dublin, Ireland) and 4 stenotic BMVs (27 mm and 29 mm, each with mild or severe obstruction) were evaluated. The imaging chamber was placed within a 1.5 T CMR scanner (Magnetom Avanto, Siemens Healthineers, Erlangen, Germany) under targeted diastolic flow conditions of 70, 90 and 110 mL at 70 beats per minute.

**Fig. 4:**
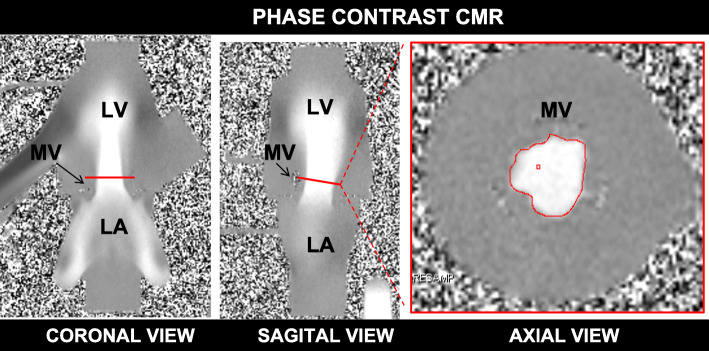
Phase contrast forward flow assessment. Phase contrast flow measurement at the valve tips of a 33 mm MV. LA = left atrium. LV = left ventricle. MV = mitral valve.

For CMR gating, a mock QRS waveform was generated from laser displacement signal to trigger the gated sequences. Conventional CMR cine assessment with balanced steady-state free precession (bSSFP) sequences provided morphological and functional data. Gadolinium contrast (20 ml; gadopentetate dimeglumine (Magnevist), Bayer Healthcare Whippany, New Jersey, USA) was also added to improve signal to noise ratio. bSSFP pulse sequences were used to assess valve anatomic orifice area (AOA). The imaging plane was placed perpendicular to the trans-prosthetic inflow jet at the level of the valve tips (Fig. 4). CMR imaging parameters were: slice thickness 2.5 mm, flip angle 90^°^, field-of-view 230 mm, bandwidth 930 Hz/px, echo time (TE) 1.53 milliseconds, in-plane spatial resolution (1.0 × 0.9 mm) and temporal resolution of 48.4 ms/frame. PC CMR quantified flow using through-plane PC velocity mapping. PC pulse sequences were used to assess transvalvular peak velocity and forward flow volume (FFV), using semi-automatic contour detection software (Argus, Siemens Healthineers). PC imaging parameters were slice thickness 4 mm, flip angle 30^°^, field-of-view 230 mm, bandwidth 592 Hz/px, in-plane spatial resolution (1.2 × 0.9 mm), acquisition time 144 s using 8 averages and temporal resolution of 48.9 ms/frame. MATLAB software (Mathworks, Natick, Massachusetts, USA) was used offline to semi-automatically calculate time velocity integral (TVI) from the trans-mitral PC peak velocities recorded for each cardiac cycle. PC-EOA was calculated by dividing the PC forward flow volume by the calculated PC-TVI.

### Transthoracic echocardiography

**Fig. 5:**
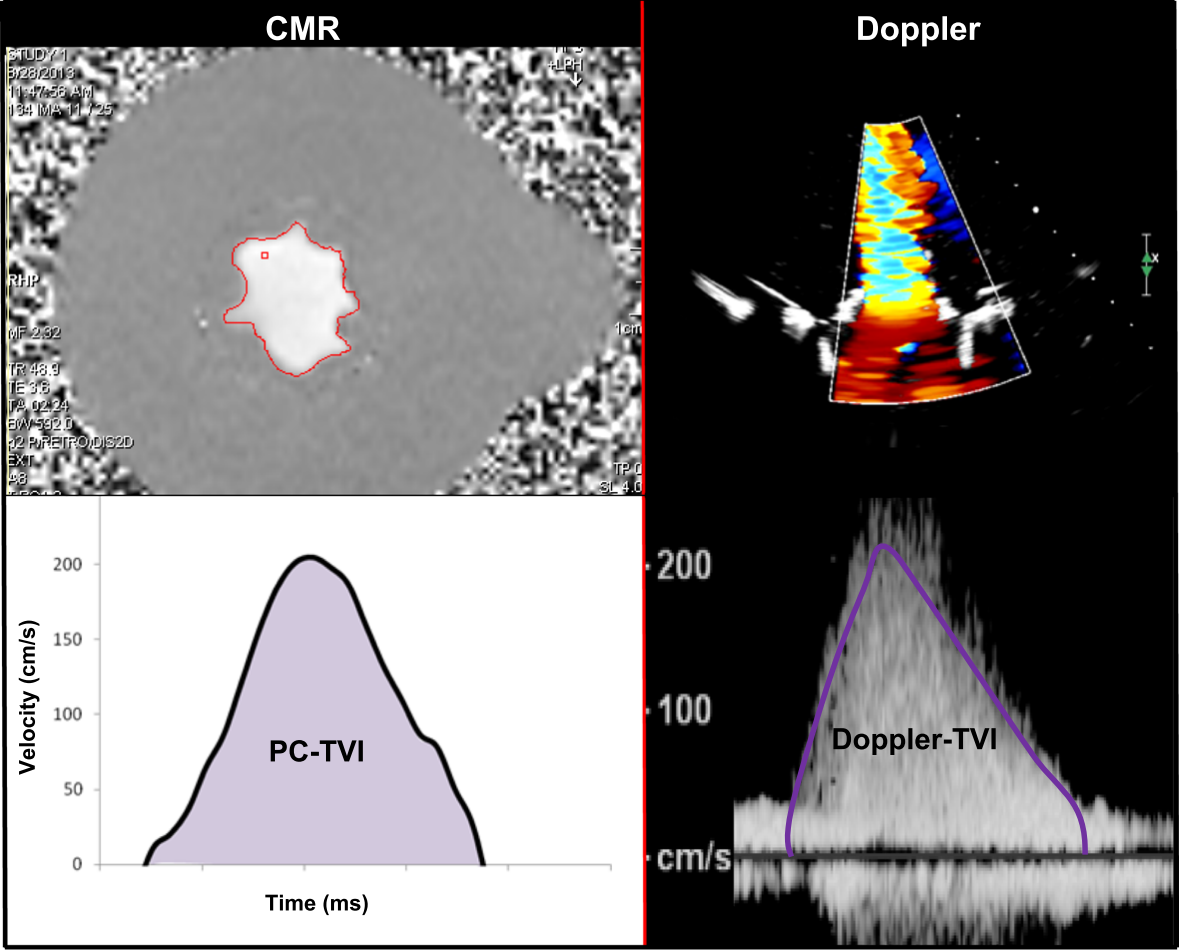
CMR and Doppler-derived time velocity integral assessment. Phase contrast image for a 31 mm BMV at a forward flow volume of 90 mL/beat. Phase contrast time velocity integral (TVI) by CMR (*left*), and spectral Doppler (*right*)

Doppler echocardiographic measurements were performed using a multifrequency (1–5 MHz) transthoracic echocardiography (TTE) probe (iE33, Philips Healthcare, Andover Massachusetts, USA). Continuous wave Doppler recording of transmitral flow was acquired from an “apical” imaging window obtaining axial alignment with transmitral inflow. Peak velocity, peak pressure gradient, mean pressure gradient and Doppler TVI were averaged from five consecutive measurements using an offline work station (Digisonics, Houston, Texas, USA). The Doppler EOA for each BMV (normal or stenotic) was calculated for each flow condition as the FFV (measured by in vitro flow meter) divided by the Doppler TVI (i.e., EOA = forward flow volume/TVI). All echocardiographic measures were performed by a separate operator blinded to CMR measurements (Fig. 5). Parameters of prosthetic valve function were assessed by CMR (including PC-TVI, PC-EOA, AOA, peak velocity and FFV by phase contrast) during 3 different flow conditions and were compared with reference standard measures (ultrasonic in vitro flow meter FFV, Doppler EOA, Doppler peak velocity and Doppler TVI).

### Statistical analysis

Mean or median values are reported for each imaging method. Correlation between observed values and reference standards was assessed using Pearson’s coefficient. The Bland-Altman [5] method was used to assess agreement between the two methods. Interobserver and intraobserver variability was assessed using a two–way random single measure and one–way random single measure intraclass correlation coefficient (ICC) analysis of all reported measures. Differences were considered significant at a *P*- value < 0.05. Statistical analysis was performed using Stata (v13.0 Stata Corporation, College Station, Texas, USA).

## Results

For a comparison of echocardiographic and CMR imaging methods we present the combined data of mean values for the normal and stenotic valve constructs (*n* = 24 test conditions). A summary of the mean hemodynamic parameters for the normal and stenotic BMV’s groups are presented in Table 1.

**Table 1:** Comparison of hemodynamic parameters for normal and stenotic valves

	**Normal (*n*** **= 12)**	**Stenotic (*n*** **= 12)**
**FFV (mL/beat)**		
***Flow Transducer***	90 ± 16	91 ± 18
***PC-CMR***	93 ± 16	88 ± 17
**Peak Velocity (cm/s)**		
***Doppler***	148 ± 33	282 ± 77
***PC-CMR***	133 ± 34	275 ± 76
**AOA (cm**^**2**^**)**		
***CMR***	2.9 ± 0.5	1.2 ± 0.3
**Mean gradient (mm Hg)**		
***Doppler***	4.0 ± 1.7	16.0 ± 8.9
***Pressure Transducer***	4.7 ± 1.0	14.8 ± 7.8
**EOA (cm**^**2**^**)**		
***Doppler***	1.8 ± 0.3	1.0 ± 0.2
***PC-CMR***	2.1 ± 0.4	1.0 ± 0.2

Forward flow volume *FFV*, Anatomic orifice area *AOA*, Effective orifice area *EOA*, Phase contrast cardiovascular magnetic resonance *PC-CMR*

### Comparison of CMR and flow transducer measures of forward flow volume

**Fig. 6:**
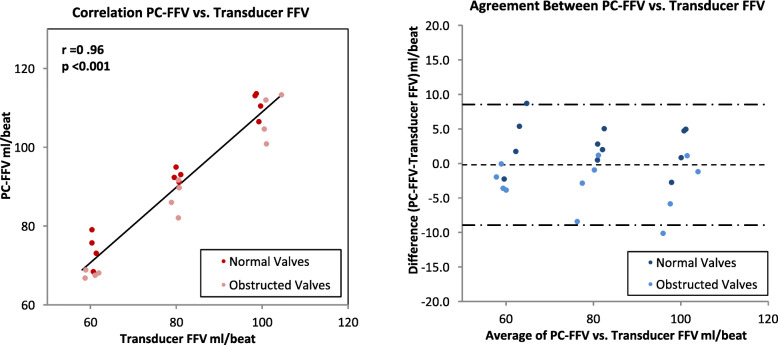
Plot of phase contrast CMR forward volume against reference volume. Pearson correlation analysis between PC- forward flow volume (PC-FFV) and flow transducer FFV (*left)*. Bland-Altman plot and limits of agreement (*right*)

PC-CMR pulse sequences were acquired and instantaneous flows were plotted throughout the diastolic filling period. PC derived median FFV was 91.4 mL/beat (*N* = 24; range 66.8 to 113.6 mL/beat) and was similar to the flow meter reference standard (median 90.5 mL/beat, range 68.8 to 114.5 mL/beat). For both normal and stenotic bioprosthetic mitral valves, PC-CMR accurately measured forward flow volume across the range of predefined reference flow volumes (*r* = 0.96, *P* < 0.001). Bland-Altman analysis of the two techniques demonstrated a 95% confidence interval from − 8.9 to 8.5 mL/beat and a clinically insignificant FFV bias of − 0.2 mL/beat for the PC-CMR method (Fig. 6).

### Comparison of CMR anatomic area and Doppler effective orifice area

The mean CMR-derived AOA was 2.1 ± 1.0 cm^2^ across all flow conditions and BMV sizes. CMR AOA correlated well with the Doppler EOA (*r* = 0.99, *P* < 0.001) however the mean Doppler EOA was substantially smaller; 1.4 ± 0.5 cm^2^. Of note, the difference between CMR AOA and Doppler EOA was largest for normal valves (38% relative difference) but was substantially smaller for the stenotic valve constructs (17% relative difference) which were associated with greater transvalvular pressure gradients for all flow volumes tested.

### Comparison of CMR and Doppler effective area

Across all conditions tested, the Doppler and PC-CMR measurement of trans-valvular velocity (median 175 vs 175 cm/s, respectively) correlated highly (r = 0.99, *P* < 0.001), with a minimum bias of − 11 cm/s, and narrow limits of agreement (− 41 to 19 cm/s). Doppler and PC-CMR measurement of TVI (74 ± 32 cm vs 71 ± 33 cm, respectively) correlated highly (r = 0.99, *P* < 0.001), with a minimum bias of − 3.8 cm, and narrow limits of agreement (− 12.3 to 4.8 cm).

**Fig. 7:**
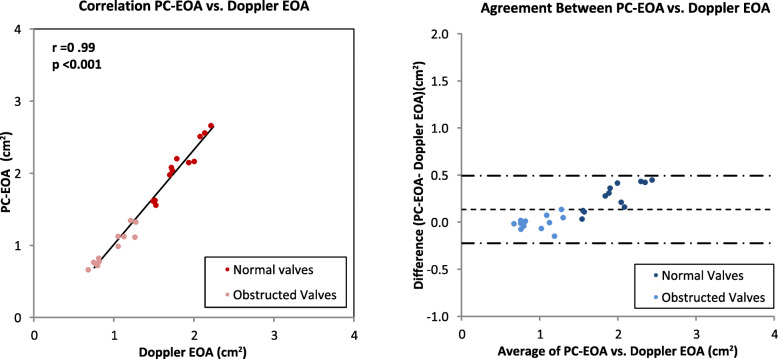
Plot of phase contrast effective orifice area against Doppler method. Pearson correlation analysis between phase contrast effective orifice area (PC-EOA) and Doppler effective orifice area (EOA) (*left*). Bland-Altman plot and limits of agreement (*right*).

Across all conditions tested, Doppler and PC-CMR measurement of EOA (1.4 ± 0.5 vs 1.5 ± 0.7 cm^2^, respectively) correlated highly (r = 0.99, *P* < .001), with a minimum bias of 0.1 cm^2^, and narrow limits of agreement (− 0.2 to 0.5 cm^2^). Since EOA is derived as volume (cm^3^)/TVI (cm), and since PC-TVI was smaller than Doppler TVI, it follows that the PC-EOA was slightly larger than the Doppler EOA for each valve construct evaluated (Fig. 7).

### Reproducibility

PC-FFV assessment demonstrated good reproducibility between observers (ICC = 0.99) and on repeat measurement by a single observer at a later date (Intrasubject variability; ICC = 0.99). The semi-automatic method of PC-TVI calculation described was also associated with good reproducibility between two observers (ICC = 0.99) and for intra-subject assessment (ICC = 0.99). In addition, PC-EOA calculated using the forward flow volume divided with TVI, yielded a good interobserver (ICC = 0.98) and intraobserver variability (ICC = 0.99).

## Discussion

In this study we used an in vitro pulsatile model to permit the parallel comparison of Doppler and PC-CMR methods for the functional evaluation of normal and stenotic bioprosthetic mitral valves. We demonstrated that phase-contrast CMR can be used to measure the diastolic EOA with values very similar to those obtained by the Doppler method across a broad range of flow conditions.

We established that an in vitro pulsatile system can be employed to assess the CMR-based functional assessment of both normal and stenotic mitral bioprosthetic valves. As noted in Table 1, the hemodynamic parameters of the normal valves are consistent with the flow volumes that would be expected clinically for a similar range of valve sizes. Specifically, the mean EOA of 1.8 cm^2^, mean gradient of 4 mmHg, and a peak velocity of 148 cm/s are well within expected range for normal bioprosthetic valves ranging in size from 27 to 33 mm [6, 7]. For the stenotic valve constructs a mean Doppler derived EOA of 1.0 cm^2^ (range 0.7–1.3 cm^2^), mean Doppler gradient of 16 mmHg (range 4–32.2 mmHg), and a mean peak Doppler velocity of 282 cm/s (range 151–418 cm/s), are all consistent with clinically encountered stenotic indices.

### Functional assessment of valves

It is well recognized that bioprosthetic heart valves undergo degenerative changes and are subject to develop stenotic or regurgitant dysfunction years after implantation [2]. Doppler echocardiography remains the imaging tool most commonly employed to assess bioprosthetic valve function. Published guidelines describe the parameters of normal and abnormal prosthetic valve function which include peak diastolic velocity, mean pressure gradient, and EOA derived from the continuity equation [1, 2]. However, for some patients, Doppler methods are technically limited due to body habitus, imaging angles, or other factors leading to unreliable data. For such patients, PC-CMR may offer an alternative imaging method to assess bioprosthetic valve function.

Although clinical use of CMR for bioprosthetic mitral valve function has been described, [8] there are no published reports describing the accuracy of CMR measurements of peak valve velocity, or pulsatile transvalvular flow rate compared to an in vitro reference standard. Such validation efforts are important since CMR methods do have some inherent limitations compared to Doppler methods. The temporal resolution of current CMR systems usually ranges between 25 and 50 milliseconds and is lower than continuous wave Doppler [9]. In clinical scanning, lower PC in plane resolution is acquired. In addition, the PC slice must be placed at the location of maximal velocity and some velocity underestimation may occur because of slice thickness partial voluming. High temporal acceleration is a key characteristic of highly pulsatile jet flow and the low temporal resolution of PC especially in the clinical setting may blunt the accuracy of the velocity time waveform. As such, peak velocity, PC-TVI can be both underestimated and PC-EOA overestimated compared to Doppler standards in clinical settings. With these potential limitations in mind, it is important to establish the accuracy of a new functional imaging technique across a range of both the normal and abnormal hemodynamic conditions.

An important result for our study is the finding that PC-CMR methods can be used to directly measure diastolic flow volume at the level of the prosthetic leaflet tips with good accuracy compared to an in vitro flow meter reference standard. Prior in vivo studies have validated the accuracy of PC-CMR methods to measure either forward or regurgitant flow volumes against reference standards [10–13]. However, these studies were not an evaluation of flow measure across prosthetic valves. Previously, Von- Knobelsdorff et al. [8] demonstrated a strong correlation (*r* = 0.94, *P* < 0.001) between Doppler EOA and CMR planimetry area in a study of 18 patients with BMV. However, in that study the EOA was calculated by the pressure half-time method, which has been previously reported to overestimate inflow area of BMVs [14]. Strohm et al. demonstrated a strong correlation between CMR measured and true anatomic area in a constant flow (non-pulsatile) phantom using fixed orifices of different size and shape [15]. The study we present is the first to report a comparison of CMR, Doppler, and in vitro standard measures of normal and stenotic bioprosthetic valve function under controlled, pulsatile flow conditions.

A novel feature of our study was the determination of trans-valvular TVI from phase contrast data. Previous studies in aortic stenosis patients [16–18] have demonstrated the feasibility of a PC-TVI method for aortic valve stenosis evaluation. Our group has previously described a CMR based method to accurately derive the EOA in patients with normal and dysfunctional bioprosthetic aortic valves [19]. Using this approach, we calculated the PC-EOA as the product of the forward flow volume divided by the derived TVI. We report a strong correlation and good agreement with the Doppler methods to derive EOA. This application of CMR velocities to derive an estimate of the prosthetic mitral valve EOA has not been previously described.

### Anatomic and effective orifice area

Although Doppler methods also provide an estimate of peak and mean trans-valvular gradient, an accurate measure of EOA is an important parameter of prosthetic valve function since it is relatively independent of stroke volume and is therefore influenced less by variation in heart rate [1].

Semi-automated planimetry of the anatomic orifice area by cine CMR provided a highly reproducible AOA measure for all valves studied under each flow condition. Although reference standards for AOA are not available, the values reported in Table 1 are consistent with the associated hemodynamic parameters recorded (normal, mild stenosis or severe stenosis) and demonstrated a strong correlation with EOA by either Doppler or PC-CMR method. However, the mean AOA was significantly larger than the mean EOA by either Doppler or PC method. This difference in area is expected due to the different nature of these two area concepts. The AOA is a single frame measurement representing a tomographic slice of a three-dimensional flow event, whereas the EOA represents the mean diastolic flow area throughout the entire filling period. As such, the EOA does not represent the true anatomic area but rather the physiologic area occupied by flow at the plane of maximal velocities [20]. The Doppler EOA results we report are similar to clinical reports for normal Medtronic Mosaic BMVs early after implantation [6, 7]. As evident in Table 1, there is a greater difference between AOA and EOA for the normal valves than for the stenotic valve constructs. Depending on the 3D nature of the valve orifice (normal size or stenotic) the flow contraction coefficient may range between 0.6–1.0. A stenotic valve with higher transvalvular pressure may force the flow to occupy relatively more of the available anatomic area, thus decreasing the magnitude of flow contraction (creating a contraction coefficient closer to 1.0) and decreasing the difference between the AOA and the EOA [20]. This finding is consistent with the hydrodynamic principal that more of the available flow area (the anatomic area) is utilized under conditions of higher pressure gradient. Since the stenotic valve constructs were associated with a 4-fold greater mean gradient, it follows that more of the available AOA is utilized under higher driving pressures. In short, a direct measurement of the AOA provides an area considerably larger than the EOA unless the transvalvular pressure gradients are significantly elevated. This observation may have important implications for the clinical interpretation of any native or prosthetic anatomic valve area assessed by CMR planimetry method. At times, AOA planimetry can be challenging even for experienced readers, especially in clinical cases with significant leaflet calcification and substantial strut artifact. In such cases, reporting PC-EOA is an alternative since is comparable to Doppler EOA to assess valve function.

**Fig. 8.**
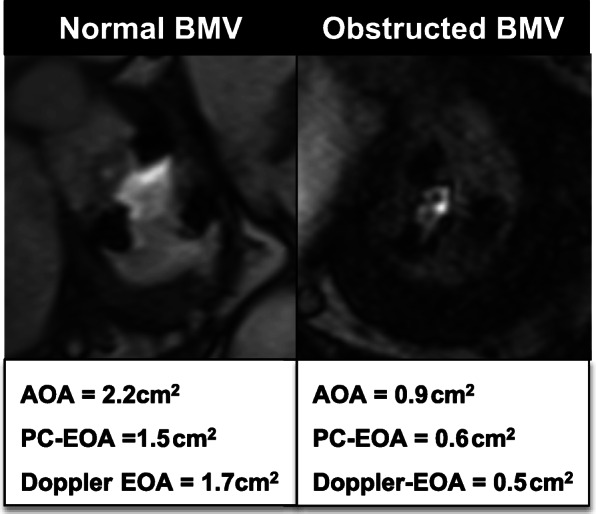
Clinical examples of normal and obstructed bioprosthetic valves assessed by CMR. A 65 year old male with a 29 mm bioprosthetic mitral valve. Phase contrast effective orifice area (PC-EOA) and DopplerEOA were similar and within normal range (left). A 60 year old male with dyspnea and a 25 mm bioprosthetic mitral valve. PC-EOA and DopplerEOA were both severity reduced, consistent with severe prosthetic valve stenosis (right)

We present a validation of CMR methods and a novel derivation of PC-EOA with comparison to Doppler standards. A similar in vivo study of the two imaging methods is difficult to perform since identical flow conditions cannot be ensured in patients during serial imaging studies. However, we provide a clinical example where both Doppler and CMR methods were employed to evaluate AOA and EOA in patients with normal and stenotic bioprosthetic mitral valves respectively (Fig. 8).

### Future perspectives

Currently, thresholds for mean gradients and EOA, described in the Mitral Valve Academic Research Consortium criteria have been used to evaluate the success of transcatheter mitral valve replacements for degenerated BMVs and failed mitral annuloplasty rings [21]. In addition to the valvular lesion assessment, CMR may evaluate the impact of valve intervention on cardiac function and remodeling. Of note, CMR provides unique information regarding the extent of myocardial interstitial and/or replacement fibrosis. It is quite obvious, that CMR may provide very important target parameters used in clinical trials and as such future studies in the clinical environment may focus in assessing the accuracy of the EOA as a measure of BMV function using this novel approach.

### Limitations

In our study we had the advantage of scanning a fixed BMV, thus avoiding the disadvantage of BMV displacement during the cardiac cycle. The extent to which this motion may or may not affect the PC assessment of BMV function has not yet been established. Motion tracking algorithms have been developed and could be utilized to mitigate these effects if clinically indicated [22, 23]. In the clinical environment some patients suffer coincident arrhythmias which can reduce the accuracy of many CMR measures. In our experimental set up, we used a single type of BMV and a silicone rubber occluder to model BMV stenosis. In the clinical setting, direct planimetry of AOA can be challenging in cases where strut artifact is substantial or if valve leaflets are heavily calcified. In our in vitro study design a single pulsatile pump was utilized to create a single phasic filling during diastole. The contribution of atrial contraction to diastole was not modeled. However, we replicated Doppler waveforms of early and mid diastolic filling. PC-TVI was calculated using peak diastolic velocity data and is not expected to be affected using a single or biphasic filling model.

## Conclusion

We describe a novel method to assess BMV function based on PC-CMR measurements of transvalvular flow volume and velocity integration. PC-CMR methods can be used to accurately measure EOA for both normal and stenotic BMV’s and may provide an important novel parameter of prosthetic valve function when Doppler methods are unobtainable or unreliable.

## Supplementary information

**Additional file 1: Video S1.** depicts in vitro video images of normal bioprosthetic leaflet motion as viewed from the left ventricle imaging window.

**Additional file 2: Video S2.** depicts in vitro video images of a stenotic bioprosthetic valve construct as viewed from the left ventricle imaging window.

**Additional file 3: Video S3.** depicts cine CMR images of normal bioprosthetic leaflet motion.

**Additional file 4: Video S4 and Video S5.** depict phase contrast images of a normal bioprosthetic valve from long- axis and short-axis views, respectively. Both views are used to ensure that flow volume is measured at the level of the prosthetic leaflet tips.

## Data Availability

The data sets used and/or analyzed during the current study are available from the corresponding author on reasonable request.

## Abbreviations

AOA: Anatomic Orifice Area
BMV: Bioprosthetic mitral valve
bSSFP: Balanced steady state free precession
CMR: Cardiovascular magnetic resonance
EOA: Effective orifice area
FFV: Forward flow volume
PC: Phase contrast
TTE: Transthoracic echocardiography
TVI: Time velocity integral
